# Isolated Tracheabronchomalacia Misdiagnosed for Years as Bronchial Asthma

**DOI:** 10.7759/cureus.35641

**Published:** 2023-03-01

**Authors:** Lubna Almogarry, Alzahra Alradhi, Abdullah S Alshamrani

**Affiliations:** 1 College of Medicine, Imam Abdulrahman Bin Faisal University, Dammam, SAU; 2 Pediatrics, King Fahad Medical City, Riyadh, SAU; 3 Pediatrics, Alfaisal University, Riyadh, SAU

**Keywords:** bronchial asthma, wheeze, tracheabronchomalacia, brochomalacia, tracheomalacia

## Abstract

Tracheomalacia (TM) is an abnormal collapse of the tracheal lumen, which often occurs when the cartilaginous part of the trachea has not developed. It is a rare condition but is seen often in infancy and childhood period. The incidence of primary airway malacia in children was estimated to be at least one in 2,100. It has a wide range of etiologies, and it is often localized but rarely generalized as in our case. It could be severe enough to indicate frequent admission and might expose the patient to multiple unnecessary medications.

We are reporting a case with unusual primary tracheobronchomalacia (TBM) that was missed for several years with a huge burden on both families and healthcare providers. A five-year-old Saudi girl had multiple admissions to the intensive care unit with similar presentation each time, and she was misdiagnosed as having asthma exacerbation with an occasional chest infection. Bronchoscopy revealed the underlying condition, and the patient was kept on the minimal intervention of nasal continuous positive airway pressure (CPAP) and aggressive airway hydration therapy, all with the goal of improving the patient's outcome and reducing hospital admissions.

We emphasize the importance of alerting physicians about malacia as an important cause of recurrent wheezy chest, which is one of the common asthma mimickers; in such cases, flexible bronchoscopy remains the gold standard diagnostic test, while the treatment remained supportive.

## Introduction

Airway malacia occurs when the airway wall collapses due to inadequate cartilage to keep the airway open during respiration, resulting in a restricted lumen and increased airway secretions [1]. Laryngomalacia, isolated tracheomalacia (TM), isolated bronchomalacia (BM), and tracheobronchomalacia (TBM) are the main types of airway malacia [1,2]. TM is a condition in which the tracheal cartilage and/or the posterior membrane have a structural impairment, causing the airway to collapse. Rarely, when the main bronchi or one of their distal branches is involved, it is called TBM [1,2]. TM and TBM have two types of causes: congenital problems associated with an abnormally compliant or collapsing airway and secondary causes leading to deformed airway cartilage [3,4]. Secondary causes include tracheoesophageal fistula, cardiac or mediastinal compression, post-intubation, post-tracheostomy, chronic infections, and inflammatory conditions [3,4]. Because the trachea and the esophagus have a similar anatomical genesis in the embryologic growth stage, congenital TM and TBM can be associated with other congenital problems such as esophageal atresia with or without tracheal-esophageal fistula [1]. The incidence of primary airway malacia in children was believed to be at a minimum of one in 2,100 while secondary forms have a variable incidence in accordance with their etiology [5]. Clinical manifestations are often non-specific and vary depending on anatomical location and severity of the pathology [1,3]. In most of these cases, symptoms appear after the second month of life, but patients with long-segment TM and TBM may present at birth [1].

Symptoms may include barking cough, expiratory rhonchi or inspiratory stridor, and shortness of breath [6]. Ineffective coughing can lead to a buildup of secretions in the airway, predisposing to upper respiratory infections and pneumonia. Symptoms might increase in severity with increasing intrathoracic pressure as the patient performs the Valsalva maneuver, coughing, crying, feeding, or lying supine [1,4]. Due to insufficient ventilation, patients may also develop exercise intolerance, hypoxic episodes, or apneic episodes [4,6]. Patients can be symptom-free by the age of two when the tracheal cartilages mature and become more rigid [3]. In addition, the presence of maternal diabetes, hypertension, and the use of steroids can worsen the manifestation of TBM [4,7].

TM and TBM are difficult to be diagnosed because of limited clear guidelines [8]. Flexible endoscopy, which includes laryngoscopy, tracheoscopy, and bronchoscopy, is the most accurate method of diagnosis; yet, three-phase dynamic bronchoscopy is the gold standard method [2,8]. The first phase is performed while the patient is taking shallow breaths to allow visualization of the anatomical structures of the airway as well as the compression site. Also, it gives details about cartilage malformation, secretion accumulation, and vocal cord motion [2,8]. The most vital step is the second phase, which is conducted while the patient is performing the Valsalva maneuver or coughing [2,8]. After aspirating all the secretions, the next phase involves extending the airways to 40-60 cm of water. The presence of a tracheoesophageal fistula (TEF) and other related abnormalities can be ruled out during this phase [2]. Expiratory central airway collapse (ECAC) is referred to as expiratory narrowing of more than 50% in the trachea and bronchus due to a variety of causes including airway malacia [2,6].

According to the degree of narrowing, it is classified as mild, moderate, or severe. Mild obstruction is described as a collapse of 51%-75%, moderate collapse is 76%-90%, and severe collapse is 91%-100% [2,6]. Although there are no set protocols for the diagnostic airway narrowing degree, in most cases, a narrowing of more than 50% in the expiratory phase in combination with a clinically obstructed airway is considered diagnostic [6]. However, this classification does not reflect the clinical severity of the disease [1,6]. Other diagnostic modalities include chest x-rays that may occasionally show mediastinal mass. Lateral neck x-ray might also show intrathoracic trachea narrowing [4]. In addition, modiﬁed dynamic computed tomography angiogram (MDCT) can be used for localization and determination of the severity of TM and TBM, and it plays an important role pre-operatively [4]. Also, an esophagram, ventilation-perfusion, and an echocardiogram should all be conducted prior to surgical intervention [1].

Structural stability is achieved after a few years without surgical intervention in most cases [1]. Airway secretions can be managed by administering ipratropium bromide and hypertonic saline or normal saline [1,4]. Pulmonary hygiene, chest physiotherapy, and control of gastroesophageal reﬂux to prevent aspiration are also recommended [1]. Steroids are usually not advised because of their risk of cartilage degradation [4]. Bronchodilators have also been shown to exacerbate symptoms by lowering the tone of airway smooth muscles [1,4]. Previously, the initial management of severe TM and TBM was established by performing a tracheostomy and placing the patient on long-term mechanical ventilation [1,3]. However, it is known that as the child grows, the tracheostomy measurements need to be adjusted frequently, given the risk of tracheal complications [1]. This report represents the case of a five-year-old girl with shortness of breath associated with a harsh dry cough and multiple admissions to the pediatric intensive care unit (PICU) due to primary TBM.

## Case presentation

A five-year-old girl was admitted to the general pediatric ward with a three-day history of shortness of breath triggered by a flu illness. The shortness of breath was associated with an intermittent dry, harsh cough that increased in severity with activity and did not improve with the use of a Ventolin nebulizer at home. There was no evidence of diurnal variation in the symptoms, and there was no cyanosis, sweating, change in the level of consciousness, or post-tussive vomiting. She also has an unremarkable neonatal and developmental history. She was diagnosed with bronchial asthma when she was one-year old and had been treated with salbutamol, fluticasone, and a montelukast with no improvement. She has a history of poor compliance due to unsatisfactory response despite good device use technique and a negative history of contact with pets or smoke exposure.

Her past medical history included a lower respiratory tract infection (LRTI) at the age of two months and subsequent admissions every two to three months for a total of eight admissions to the PICU and 18 admissions to the general pediatric ward. All of her past admissions were treated as asthma exacerbations precipitated by upper respiratory tract infection (URTI), and she was discharged in a good condition with asthma medications.

On examination, she appeared unwell with severe respiratory distress, no finger clubbing or dysmorphism was noticed. She had a respiratory rate of 48 breaths/minute, pulse rate of 135 bpm, blood pressure of 114/69 mmHg, and a temperature of 38.5. Oxygen saturation ranged from 86% on room air and 93% on oxygen 0.5 l/m, and she had normal growth parameters. Auscultation of the chest revealed reduced breath sound on the left lower side with a predominant wheezy chest. An ejection systolic murmur in the left upper sternal border, grade 3/6, was discovered during a cardiovascular system evaluation. Other examinations were unremarkable.

**Figure 1:**
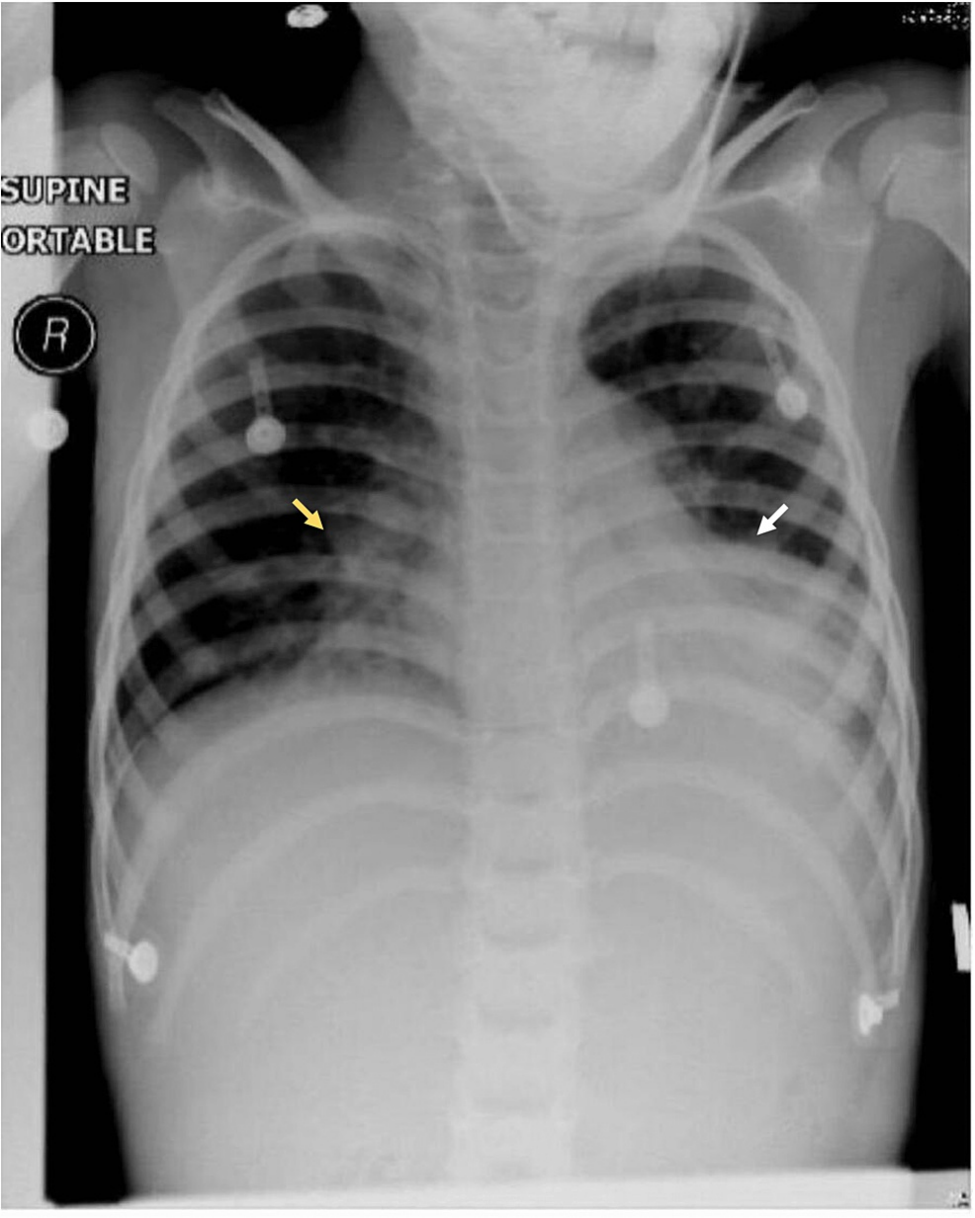
Plain chest x-ray, anteroposterior view, showing opacity in the left lower zone with air bronchogram silhouetting the cardiac border and cardiophrenic border (white arrow) and right-sided peribronchial wall infiltrate (yellow arrow), with compensatory mild hyperinflation

The initial blood investigations were unremarkable; however, the initial venous blood gas readings revealed compensated respiratory acidosis (pH 7.37, PCO_2_ 55, HCO_3_ 21 mmHg). Chest x-ray showed a significant collapse of the left lower lobe, right-sided peribranchial wall infiltrate, and mild compensatory hyperinflation (Figure 1).

**Figure 2:**
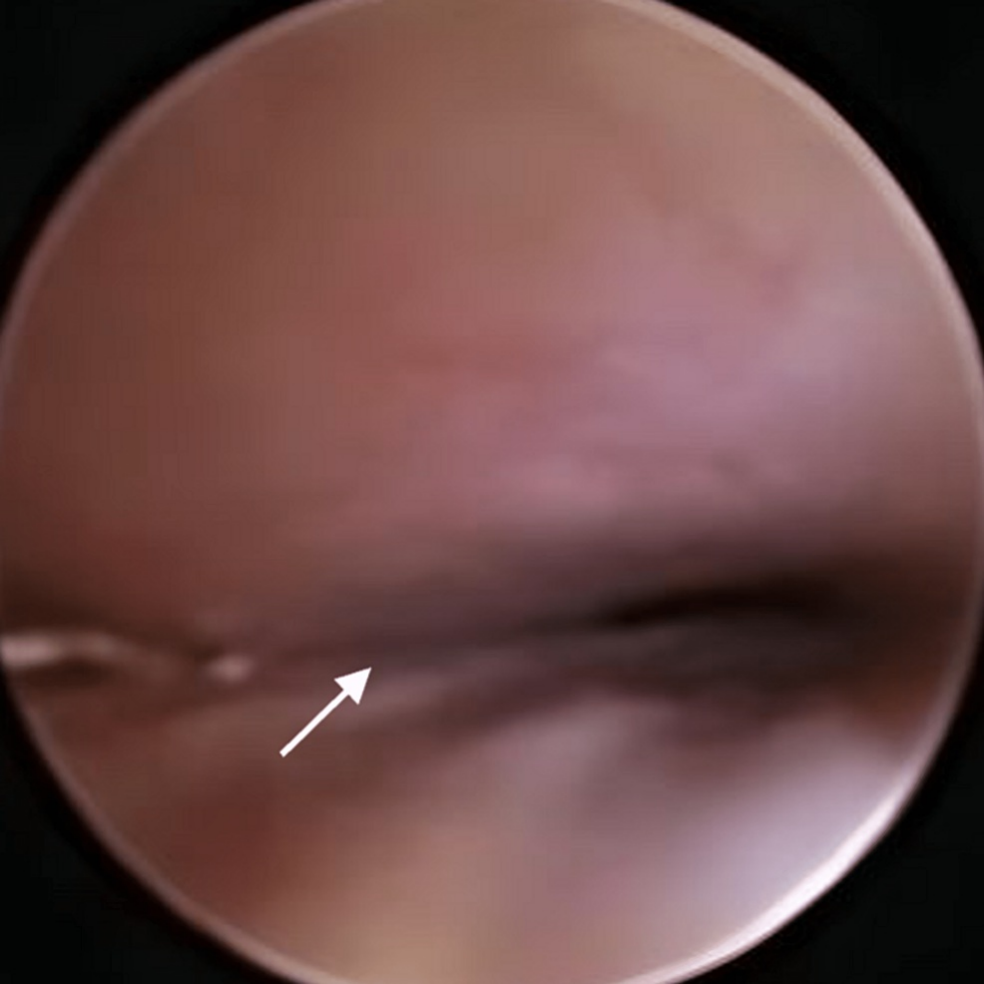
Flexible bronchoscopy at mid trachea showing severe narrowing exceeding 90% with the absence of tracheal cartilage (white arrow)

**Figure 3:**
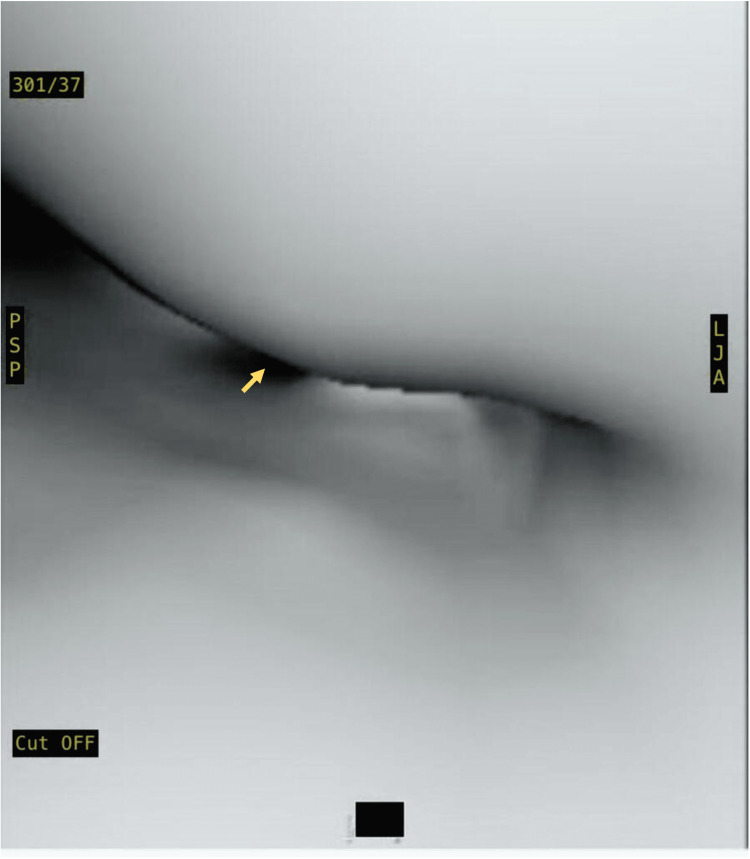
Virtual bronchoscopy at mid trachea showing severe narrowing exceeding 90% with the absence of tracheal cartilage (yellow arrow)

In addition, the barium study was positive for mild reflux. Laryngoscopy was performed with no evidence of laryngomalacia, aspiration, or other anomalies. Eventually, flexible bronchoscopy showed severe TBM with evidence of tracheal bronchus (Figure 2), and similar findings of severe TM were confirmed by virtual bronchoscopy (Figure 3).

The patient was initially admitted under general pediatrics to be treated as a case of asthma exacerbation and to rule out lower left lobe sequestration. She was started on salbutamol, steroid, clindamycin, and cefuroxime with no improvement. The patient experienced severe respiratory distress during the admission and was transferred to the PICU. Management was continued as asthma exacerbation, and no indication of intubation was encountered during her stay in the PICU. Afterward, asthma was excluded clinically due to persistent symptoms with no response to asthma medications, along with negative eosinophils and negative history of atopy. Gastroesophageal reflux disease (GERD) was considered. Foreign body aspiration was ruled out due to negative history of choking and negative signs of chest asymmetry. A sweat test was done to rule out cystic fibrosis, which was found to be negative. In addition, the patient had no history of ear or sinus infections as well as no family history of ciliary dyskinesia.

Radiological findings of opacity in the left lower zone raised the possibility of segmental collapse or a sequestrated lobe. However, there were no feeding vessels to the left lower lobe. Eventually, an airway anomaly was considered and confirmed by bronchoscopy combined with computed tomography (CT) scan. Pediatric pulmonology was consulted throughout her stay in the PICU, and it was recommended that she be transferred to a regular ward after her condition had stabilized. Afterward, there was no apparent pulsatile airway raising the possibility of vascular comparison. The thoracic surgery team was consulted and agreed on conservative management. The patient was proven to have GERD and was kept on reflux therapy. Subsequent follow-up showed marked improvement with reflux therapy, chest physiotherapy, occasional antibiotic therapy in case of infection, and continuous positive airway pressure (CPAP) when indicated.

## Discussion

This child presented with shortness of breath associated with a dry cough that was not responding to asthma management protocol, and the causes of persistent respiratory distress were investigated; she underwent bronchoscopy and CT scan of the chest, which confirmed the diagnosis of primary TBM. This case highlights the importance of considering possible congenital pathology in such cases as patients might present with acute life-threatening events.

TM is when the tracheal lumen collapses abnormally, whereas BM is when the collapse is limited to one or both mainstem bronchi and/or their divisions. They may be associated, and this condition is known as TBM [9]. Anatomically, the distal third of the trachea is the most commonly affected part in TM especially in tracheal fistula cases [1]. In our case, the narrowing was a long segment of the tracheal lumen exceeding 90% but much less involved at the main bronchi. The cause might be attributed to an anatomical abnormality or to a secondary etiology compressing the airway [3]. Isolated TM and TBM can be a manifestation of some syndromes including chromosomal defect syndromes, mucopolysaccharidoses, and inherited connective tissue disorders [9]. However, no genetic study was indicated in our patient.

Clinically, depending on the location and the severity of the defect, patients may have a variety of symptoms [3,10]. Stridor might be a manifestation of extra-thoracic trachea involvement, whereas monophonic wheezing is often found if the intrathoracic trachea is involved as our patient had a predominantly wheezy chest in presentation [10]. Patients frequently present with signs of noisy breathing, barking cough, and harsh wheezing before the age of six months, which are usually seen when the patient has an URTI [1]. In most cases, gastroesophageal reflux is considered; nevertheless, wheezing does not respond to anti-reflux medication [4,9]. Severe respiratory distress, paroxysmal cough, and apneic episodes are also likely in severe cases [3,4]. Patients may also have "death spells," which are periods of cyanosis and hypoxia that cause unconsciousness but eventually lead to the airway reopening due to hypotonia [3,4,9].

An eight-month-old kid with a history of cough, wheezing, respiratory distress, and repeated acute life-threatening episodes showed a similar presentation to our case. After bronchoscopy, the patient was diagnosed with bilateral BM and was treated with CPAP [11]. A 10-week-old baby girl with stridor, on the other hand, had a different presentation. She was later diagnosed with mainstem bronchial collapse as well as the involvement of the distal trachea due to tracheal rings [12]. Physical examination, in most cases, will be negative during inspiration because of the insignificant diameter change during this stage. However, patients experience wheezing upon forced expiration due to airway collapse in the affected portion of the bronchus [1,9]. Wheezing is often monophonic, harsh, and low-pitched on physical examination [9].

TBM can be a challenging diagnosis due to its unspecific signs and symptoms that might be misdiagnosed as asthma in some cases [12]. MDCT is a new diagnostic modality in the assessment of TM and TBM with high sensitivity and specificity (96.3% and 97.2%, respectively) [10]. This diagnostic approach is beneficial in the evaluation of mediastinal and vascular anomalies in addition to distal airway evaluation [10]. This modality demands a specific technique in which the patient must follow breathing directions during the exam. As a result, patients under the age of five frequently require intubation to perform the test [13]. Flexible bronchoscopy is the gold standard for diagnosing airway malacia and a great tool as it has an acceptable safety profile with rare, reported cases of long-lasting or life-threatening complications [10]. Spirometry can be helpful and used as supportive evidence for airway obstruction in cooperative cases, which often shows variable intrathoracic obstruction; however, our patient failed to do the test due to her young age [14].

A case series published in 1997 presented 17 patients with the diagnosis of primary BM. All included patients were diagnosed with reactive airway disease or gastroesophageal reflux or both [9]. Also, all patients were not responsive to bronchodilators as in our case. The main symptoms were chronic wheezing and large airway obstruction [9]. One patient required aortopexy that did not improve the obstruction [9]. There are no clear definitive management protocols, and therapy remains highly challenging [4]. Mild symptoms are managed conservatively as malacia usually improves with age [1]. Bronchodilators are usually used with caution due to their effect on reducing tracheal muscle tone [1-3]. CPAP may be administered as a long-term management option for patients with severe obstruction resulting in life-threatening episodes as well as during acute illnesses needing intensive care. Antibiotics as well as physiotherapy based on end-expiratory pressure techniques can be used to treat recurrent respiratory infections [15]. The most common successful surgical method is aortopexy, which is usually beneficial in severe cases of TBM [15], as in our patient; however, our patient had a long-segment TBM, so stenting the airway which could be considered for a localized or short segment has no role. For the same reason, tracheal resection is also not an option. While the pexy procedure could be the most feasible in this case, whether aortopexy or tracheopexy, we deferred this option at this stage as we did not observe a pulsatile airway [1]. Tracheal resection is a potential option for selected cases with short-segment TM, while tracheostomy is not an option nowadays [15,16].

## Conclusions

Primary TM is very rare in the pediatric population, while secondary malacia is not an uncommon diagnosis. TM remains an important asthma mimicker with the finding of a predominant wheezy chest on examination. Pediatricians should be alert about this condition, and bronchoscopy is the golden diagnostic test. Treatment is mainly supportive; however, nasal CPAP can help in maintaining airway patency, while surgical intervention is limited for severe cases.

## Appendices

**Table 1:** Abbreviations and their expansions

Abbreviations	Expansions
TM	Tracheomalacia
BM	Bronchomalacia
TBM	Tracheabronchomalacia
TEF	Tracheoesophageal fistula
ECAC	Expiratory central airway collapse
MDCT	Modiﬁed dynamic CT angiogram
PICU	Pediatric intensive care unit
LRTI	Lower respiratory tract infection
URTI	Upper respiratory tract infection
GERD	Gastroesophageal reflux disease
CT	Computed tomography
CPAP	Continuous positive airway pressure
